# Supplementary figures and images for: Bioengineering of a Lactococcus lactis subsp. lactis strain enhances nisin production and bioactivity
Source: PLoS One. 2023 Apr 10;18(4):e0281175. doi: 10.1371/journal.pone.0281175 (PMC10085027; doi:10.1371/journal.pone.0281175)

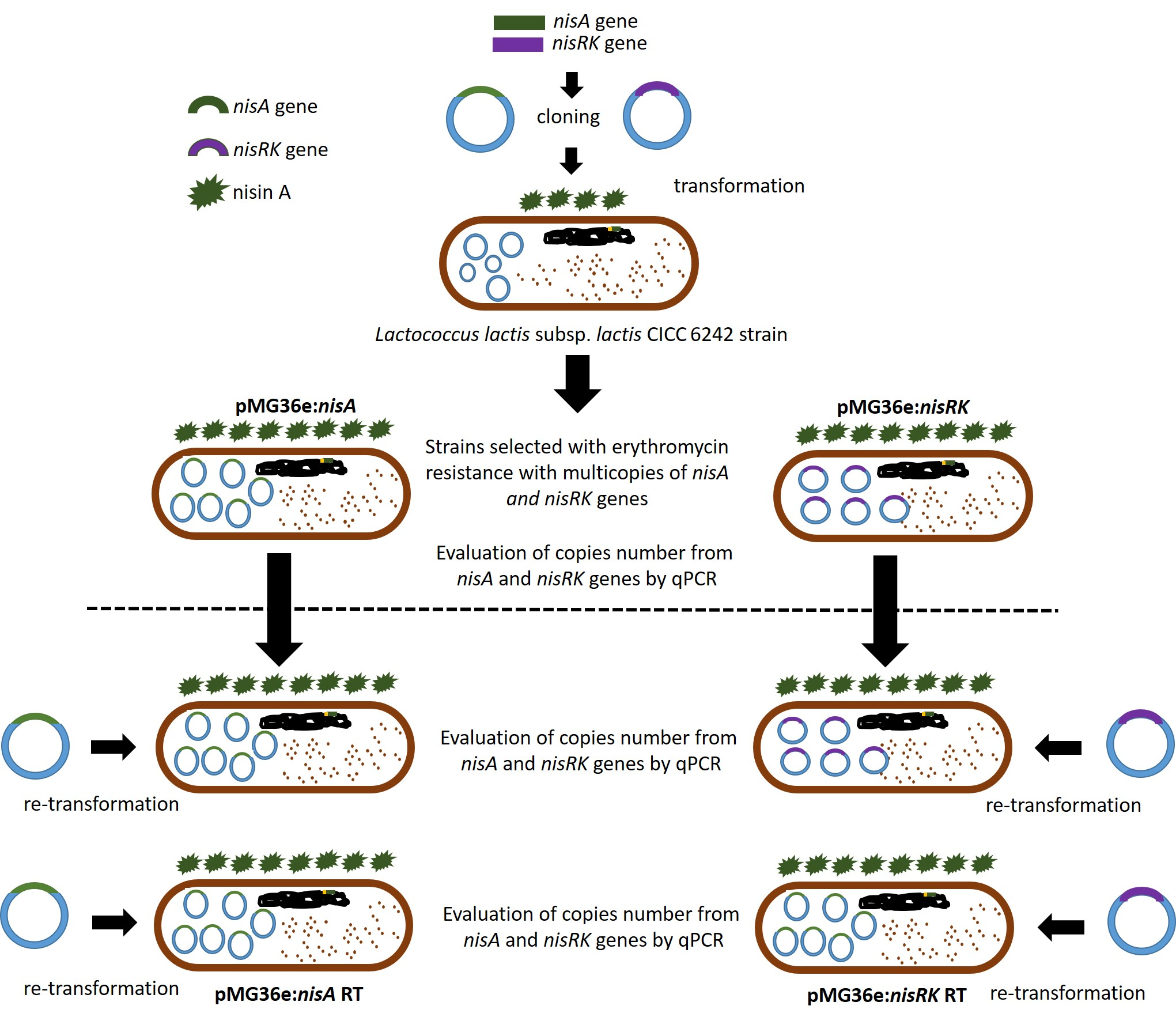

Supplement: S1 Fig — (TIF) [file pone.0281175.s001.tif]

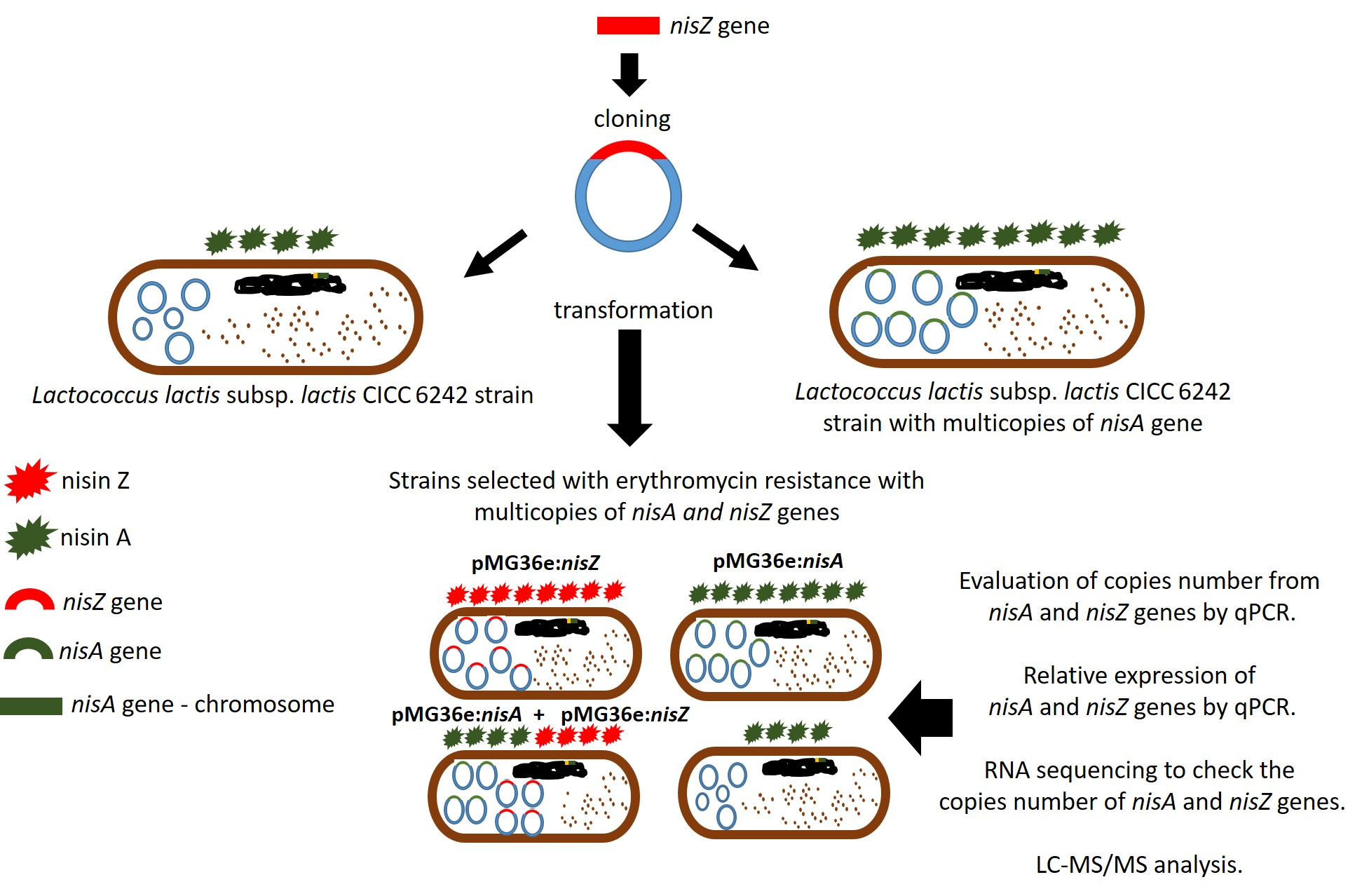

Supplement: S2 Fig — (TIF) [file pone.0281175.s002.tif]

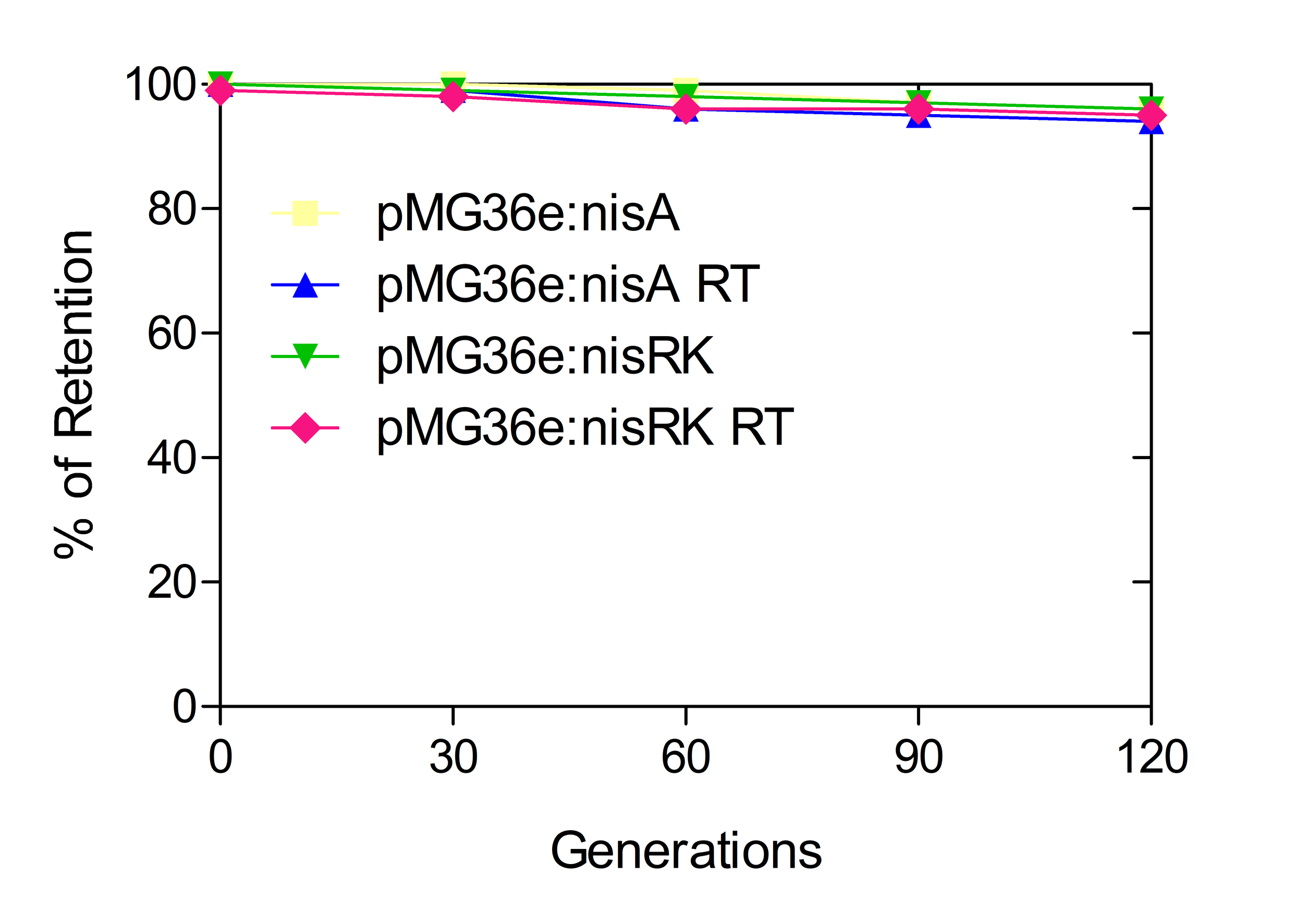

Supplement: S3 Fig — (TIF) [file pone.0281175.s003.tif]
