# Supplementary material for: Bioengineering of a Lactococcus lactis subsp. lactis strain enhances nisin production and bioactivity
Source: PLoS One. 2023 Apr 10;18(4):e0281175. doi: 10.1371/journal.pone.0281175 (PMC10085027; doi:10.1371/journal.pone.0281175)
Supplement: Attachment — Submitted filename: PlosONE-review.pdf [file pone.0281175.s004.pdf]

General comments:

The study explores the genetic transformation of *L. lactis subsp. lactis* wild-type strain to increase nisin production. Specifically, the study focuses on the overexpression of the *nisA* and *nisRK* genes in *L. lactis subsp. lactis* wild-type strain and evaluates the impact of these transformations on cell growth, nisin production, and gene expression. The study also compares the nisin productivity of the original strain and the engineered strains, including those that underwent serial genetic re-transformations. The study also shows that the co-expression of these two bacteriocins results in extremely effective antibacterial action against *M. luteus*. Overall, the findings of the study demonstrate the potential of genetic engineering to enhance the production of nisin and provide insights into the regulation of the genes involved in nisin biosynthesis. In general, quality of paper in terms of study design, and discussion of the results is fairly good.

Below are my comments and suggestions to help improve the quality of this manuscript before it could be considered for publication.

1. The study provides valuable insights into the culture conditions, preparation of recombinant strains, and gene expression strategies for the recombinant expression of nisin in Lactococcus lactis subsp. lactis. The study could be useful for further optimization of the expression of nisin and other antimicrobial peptides in *Lactococcus lactis.*
2. This study focused on increasing the expression of the *nisA* and *nisRK* genes to increase nisin production, but there may be other genes involved in nisin biosynthesis that could be targeted to further enhance production. Additional studies could investigate other genes involved in nisin production and their potential for increasing production.

3. There are few studies indicating the potential toxicity of nisin for topical use such as "In vitro assessment of skin sensitization, irritability and toxicity of bacteriocins and reuterin for possible topical applications". Therefore authors should explore if there is any research/concern regarding the potential toxicity or allergenicity of nisin in human.

4. This study showed that co-expressing nisin A and nisin Z resulted in a potentiation of activity against *M. luteus* only. It would be interesting to conduct additional antibacterial tests to evaluate the activity against other bacterial strains.

5. Please update the paper with latest references. There is only one reference from year 2022, while no reference from year 2023.
